# Differential responses of fine root biomass to nitrogen deposition mediated by functional diversity

**DOI:** 10.3389/fpls.2026.1884380

**Published:** 2026-08-12

**Authors:** Yanzhu Su, Lei Ma, Yiwen Qin, Jinhao Wang, Xinyu Wang, Dongli Wang, Lulu Wu, Chunyu Shen, Wen Li, Shenglei Fu

**Affiliations:** 1 College of Geographical Sciences, Faculty of Geographic Science and Engineering, Henan University, Zhengzhou, China; 2 National Observation and Research Field Station of Forest Ecosystem at Henan, Zhengzhou, China; 3 Key Laboratory of Geospatial Technology for Middle and Lower Yellow River Regions (Henan University), Ministry of Education, Kaifeng, China; 4 Xinyang Academy of Ecological Research, Henan University, Xinyang, China

**Keywords:** community-weighted mean, fine root biomass, functional diversity, mass-ratio hypothesis, niche complementarity, nitrogen addition

## Abstract

**Introduction:**

Fine root functional diversity can help explain and predict ecosystem functioning by integrating multiple fine root traits. However, because different functional diversity metrics emphasize distinct aspects of trait variation, their explanatory and predictive power may vary. Under ongoing atmospheric nitrogen (N) deposition, the responses of fine root functional diversity to N inputs and its contribution to community-level fine root biomass remain insufficiently understood.

**Methods:**

We conducted coordinated field experiments in warm-temperate and subtropical forests, applying three N addition levels (0, 25, and 50 kg ha^-1^ yr^-1^) and two N addition ways (canopy addition N, CAN; and understory addition N, UAN). During both the growing and non-growing seasons, we quantified community-level fine root biomass and evaluated the relative contributions of the community-weighted mean (CWM) and functional diversity (FD) of dominant species to biomass variation.

**Results:**

Our results showed that N addition increased community-level fine root biomass in the warm-temperate forest during the growing season, whereas no clear pattern emerged in the non-growing season; by contrast, fine root biomass in the subtropical forest decreased in both seasons. In both forest types, fine root biomass during the growing season was primarily explained by CWM, supporting the mass-ratio hypothesis, whereas biomass patterns in the non-growing season were better explained by FD, consistent with the niche complementarity hypothesis.

**Discussion:**

These findings highlight the pivotal role of climatic differences in mediating the effects of N addition on fine root biomass and trait expression, providing empirical evidence for understanding the applicability of the mass-ratio and niche complementarity hypotheses to belowground forest responses to N deposition.

## 1 Introduction

Over the past century, human activities such as fossil fuel combustion and agricultural intensification have increased global emissions of reactive nitrogen (N) more than an order of magnitude, resulting in atmospheric N deposition levels that far exceed natural background rates (Galloway et al., 2008; Fowler et al., 2013; Chen et al., 2025). Atmospheric N deposition has now become a major external driver of the structure and functioning of terrestrial ecosystems (Luo et al., 2025b), and exerts profound effects on plant communities and ecosystem carbon (C) cycling (Yu et al., 2019). Forest ecosystems, as major terrestrial C sinks, play critical roles in climate regulation and biodiversity conservation (Rahman et al., 2021; Verheyen et al., 2024), yet they are increasingly subjected to substantial N enrichment. The traditional “N limitation hypothesis” posits that plant growth is constrained by N availability and that N additions can enhance growth rates and increase forest net primary productivity (Lu et al., 2021). However, as N deposition has intensified, this linear expectation has been increasingly challenged (Wallace et al., 2007; Tian et al., 2016). When long-term N inputs exceed both biological demand and abiotic retention capacity, N saturation may occur, causing N loads to exceed the critical thresholds of forest ecosystems and leading to soil acidification, the leaching of base cations (Ca^2+^, Mg^2+^), and aluminum toxicity (Fang et al., 2009; Jonard et al., 2011; Xie et al., 2021).

To better understand how forests respond to N deposition across contrasting climatic conditions, community-level response strategies may be inferred from plant functional traits (Díaz et al., 2013; Funk et al., 2017). Plant functional traits provide key insights into how species acquire resources and cope with environmental stress, and they play fundamental roles in regulating biodiversity and ecosystem functioning (Díaz and Cabido, 2001; He et al., 2023). Two major hypotheses have been proposed to explain the functional role of traits in communities: the mass-ratio hypothesis and the niche complementarity hypothesis (Tilman et al., 1997; Grime, 1998). According to the mass-ratio hypothesis, dominant species, through their strong environmental adaptability and rapid resource acquisition, exert disproportionate influences on community stability and forest productivity (Baert et al., 2018; Zhang et al., 2025a). In contrast, the niche complementarity hypothesis proposes that ecosystem functioning is driven not by dominant species alone but by the degree of trait differentiation and complementarity among species (Loreau and Hector, 2001). Higher functional diversity enables species to partition resources more completely and efficiently, thereby allowing each species to realize its functional potential (Aoyama et al., 2023). These two hypotheses are not contradictory; rather, they represent complementary mechanisms through which communities maintain ecosystem functioning. Specifically, the mass-ratio effect can be quantified using the community-weighted mean (CWM), which captures the central tendency of community trait values. CWM reflects the functional dominance and trait plasticity of abundant species and represents the community’s overall resource-acquisition strategy, thereby influencing ecosystem stability through its effects on resistance and resilience (Garnier et al., 2004; Díaz et al., 2007; Garnier et al., 2007). Niche complementarity is commonly represented by functional diversity (FD) (Tilman et al., 1997; Griffin et al., 2009), which enhances ecosystem stability by promoting resource partitioning, asynchronous species responses, and insurance effects under environmental perturbations (Rao, 1982; Schnabel et al., 2021). Under increasing N deposition, the roles of FD and CWM in belowground processes remain far less explored than those of aboveground traits (Suonan et al., 2023), resulting in a limited understanding of how belowground trait responses shape community-level dynamics.

Nutrient and water uptake by belowground root systems are key process coupling nutrient cycling and energy flow in forest ecosystems (Bassirirad, 2000; Craig et al., 2025), whereas fine root (diameter, Dia ≤ 2 mm), although accounting for only 0.5%-10% of total forest biomass (Huang et al., 2024), contribute more than 30% to annual terrestrial net primary productivity (McCormack et al., 2015). Unlike coarse roots, which primarily function in structural support and storage, fine roots serve as essential organs for resource acquisition (Hendrick and Pregitzer, 1992). Because they contain abundant thin-walled cells and exhibit low lignification, fine roots generally have short lifespans and rapid turnover, which make them highly sensitive and responsive to environmental change (McCormack et al., 2015), thereby influencing terrestrial C and N cycling. The spatial distribution and dynamic patterns of water and nutrient uptake in fine roots represent important adaptive responses to environmental change (Li et al., 2025a). Morphological traits such as specific root length (SRL) and specific root area (SRA) are commonly used to quantify the potential absorptive area per unit biomass; root tips and forks reflect the intensity of nutrient foraging and soil exploration, as well as the absorptive potential of root systems (Eissenstat et al., 2015; Li et al., 2021), whereas root tissue density (TD) and Dia are associated with fine root physiology and indicate plant resource-use efficiency (Birouste et al., 2014). These traits do not necessarily respond uniformly to N inputs; plants may shift along an acquisitive-conservative trait axis (Gao et al., 2023), or exhibit trait trade-offs or compensatory adjustments (Yu et al., 2025). This complexity further suggests that integrating fine root trait information at the community-level provides greater explanatory power than focusing on single species or individual traits.

Evidence from aboveground studies suggests that the two hypotheses are not mutually exclusive. Instead, they can operate simultaneously within plant communities, with their relative shifting dynamically in response to changes in environmental conditions and community structure. In tropical forest ecosystems, the niche complementarity effect dominates during early successional stages. As stand structure matures, the mass-ratio effect gradually replaces complementarity as the primary driving mechanism (Souza da Costa et al., 2025) Conversely, in subtropical forests, productivity is mainly driven by the mass-ratio effect in early succession, whereas the niche complementarity effect prevails in mid-to late-successional stages (Wang et al., 2026b). Nonetheless, the two effects do not necessarily operate in opposition to one another. Chiang et al. (2016) demonstrated in subtropical forests in Taiwan, China, that the mass-ratio effect directly governs aboveground biomass, whereas the niche complementarity effect indirectly promotes productivity by increasing stem density. This finding indicates that the two effects can function synergistically within the same ecosystem. However, existing studies have largely focused on individual forest ecosystems, and their assessment timeframes have mostly been restricted to the growing season or annual scales (Ali et al., 2017; Hao et al., 2020; Gao et al., 2024). To date, no systematic evaluation has been conducted of the relative contributions of these two hypotheses to community-level fine root biomass across forests in different climatic zones and during distinct plant growth periods.

Another often overlooked yet critical question is how atmospheric N enters forest ecosystems. Under natural N deposition, a substantial proportion of N is first intercepted by the forest canopy, including retention on stems and leaves and uptake by epiphytic organisms (Matson et al., 2014; Nair et al., 2016), whereas the fraction that is not retained or absorbed is transferred to the forest floor through gravitational deposition or rainfall wash-off. Thus, the canopy acts as a filtering layer during the N deposition process, potentially reducing the amount of N that reaches understory soils and biota. In contrast, conventional N addition experiments apply N directly to the forest floor, which may overestimate belowground responses to N deposition relative to those under actual atmospheric N deposition. Moreover, forests across climatic zones differ markedly in nutrient status, climatic and soil conditions, and species composition (Campioli et al., 2012; Machar et al., 2017). For example, warm-temperate forests with low ambient N availability are typically considered N-limited, and N additions tend to increase fine root biomass in these systems, whereas in tropical and subtropical forests experiencing N saturation and P limitation, N inputs often have the opposite effect (Li et al., 2023). Although numerous N addition experiments have examined fine root responses in warm-temperate and subtropical forests, systematic cross-climatic comparisons remain scarce. In addition, the impacts of N deposition depend on both input pathways and forest type, highlighting the need for a better understand how N deposition affects forest ecosystems under contrasting environmental contexts.

To address the above limitations, we conducted a 9-year N addition experiment in warm-temperate and subtropical forests in China to examine the effects of different N addition regimes and concentrations on community-level fine root biomass and trait diversity. Our aims were as follows: (i;) to compare the spatiotemporal variation in fine root biomass under N addition between warm-temperate and subtropical regions; (ii) to elucidate, across climatic zones, the relative contributions of FD and CWM to community-level fine root biomass in the growing and non-growing seasons. The results of this study are expected to improve our understanding of the dominant mechanisms driving changes in community-level fine root biomass under N deposition and to clarify their applicability across climatic zones. This work may have important implications for optimizing plant community structure and enhancing ecosystem services in a changing global environment.

## 2 Materials and methods

### 2.1 Study sites

The study was conducted in two geographical regions in China: the Jigongshan National Nature Reserve (JGS) in central China and the Subtropical Shimentai National Nature Reserve (SMT) in the southern China. JGS is located in Xinyang, Henan Province (31°46′-31°52′N, 114°01′-114°06′E), and covers 2,917 ha. It has a warm-temperate monsoon climate with distinct seasonality, and most precipitation occurs during the warm season. The mean annual temperature is 15.2°C and annual precipitation is 1,118.7 mm. The soils are classified as brownish-yellow soils. The forest is classified as an evergreen and deciduous broad-leaved mixed forest, with a mean tree height of 21.6 m, a mean leaf area index of 4 m^2^ m^-2^, a canopy closure of 93%, a stand density of 446 stems ha^-1^, and a stand age of 50 years. The dominant canopy tree species are *Liquidambar formosana*, *Quercus acutissima*, and *Quercus variabilis*. The dominant shrub species include *Celtis sinensis*, *Acer buergerianum*, *Lindera glauca*, *Ilex cornuta*, and *Ligustrum quihoui*. The Herbaceous layer includes *Eriophorum scheuchzeri*, *Arthraxon hispidus* and *Vitis heyneana* (Zhang et al., 2015; Yang et al., 2024). The Current annual atmospheric wet N deposition rate at JGS is estimated to be approximately 20 kg N ha^–1^ yr^-1^ (Li et al., 2025b). The SMT site is located in Qingyuan, Guangdong Province, China (24°22′-24°30′N, 113°05′-113°30′E), and covers a total area of 33,555 ha. Situated on the northern fringe of the Tropic of Cancer, the site has a subtropical monsoon climate characterized by abundant sunlight, warm and humid conditions, and plentiful rainfall. The mean annual temperature is 20.9 °C, mean annual precipitation is 1,182 mm, and relative humidity is consistently high. The soils are classified as red soil, and the forest is subtropical evergreen broad-leaved forest, with a mean tree height of 13.8 m, a stand density of 818 stems ha^-1^, and a stand age of 55 years. The dominant canopy tree species include *Schima superba*, *Castanea henryi*, *Engelhardia roxburghiana*, and *Machilus chinensis*. The dominant shrub species are *Symplocos ramosissima*, *Blastus cochinchinensis*, and *Aidia canthioides*. The herbaceous layer is dominated by *Alpinia oblongifolia*, *Cibotium barometz*, *Dicranopteris pedata*, and *Imperata cylindrica* (Zhang et al., 2015). The mean annual wet N deposition rate at SMT was 35 kg N ha^-1^ yr^-1^ over the period 2016-2019 (Tian et al., 2022).

### 2.2 Experimental design

**Figure 1:**
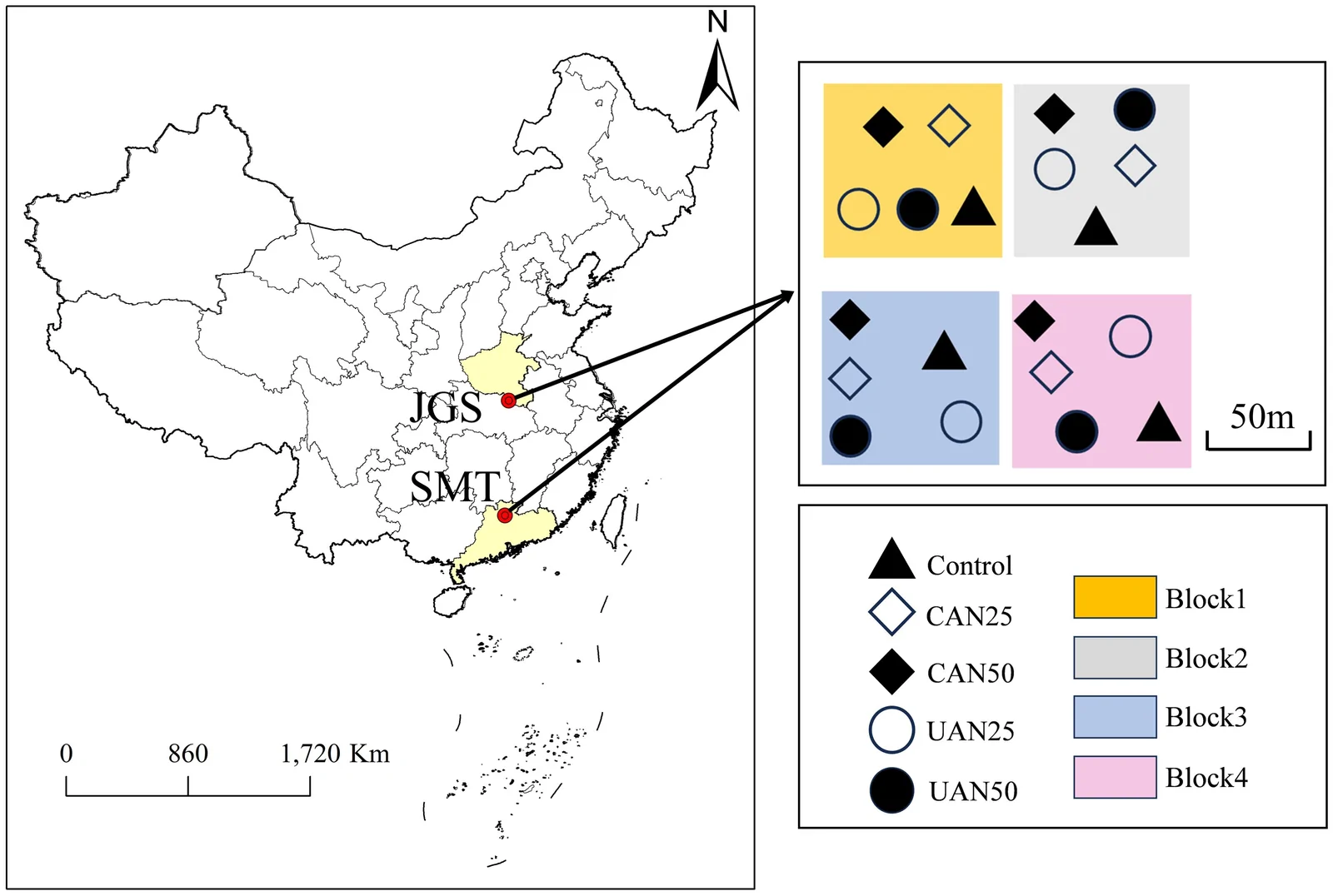
Experimental site and the locations and layout of each treatment.

The experimental treatments at both sites were initiated in 2012 and followed identical protocols. The design comprised two N addition ways (canopy addition N, CAN and understory addition N,UAN) and three N addition levels (0, 25, and 50 kg N ha^-1^ yr^-1^), resulting in five treatments: control (CK), canopy addition N of 25 kg N ha^-1^ yr^-1^ (CAN25), canopy addition N of 50 kg N ha^-1^ yr^-1^ (CAN50), understory addition N of 25 kg N ha^-1^ yr^-1^ (UAN25), and understory addition N of 50 kg N ha^-1^ yr^-1^ (UAN50). These treatments were randomly assigned to four blocks with comparable soil, topographic, and moisture conditions, yielding a total of 20 experimental plots. Each plot was circular with a radius of 17 m, covering an area of 907 m^2^ (**Figure 1**). To ensure plot independence, a 20 m buffer zone was established between plots, with a 1 m deep PVC barrier installed at the center of each buffer. At the SMT site, however, equipment failure in one plot resulted in only three replicates for the CAN25 and UAN25 treatments.

A 35 m iron tower, extending 5–8 m above the forest canopy, was constructed in each CAN plot, with a 360° rotating sprinkler installed at the top. In each UAN plot, an automated device equipped with a similar 360° rotating nozzle was installed at a height of 1.5 m above the ground. The N solution was evenly applied across each plot using these devices. The N solutions were prepared by dissolving 99.9% pure NH_4_NO_3_ in locally collected lake water and rainwater, and N concentrations were continuously monitored to ensure consistency with the target levels. Based on local phenology, N addition was conducted seven times per year from April to October, with each application performed on calm, clear mornings or evenings. The amount of solution applied during each event was equivalent to 3 mm of precipitation. The N addition treatment introduced approximately 21 mm of additional water per year along with the NH_4_NO_3_ solution, whereas the CK did not receive an equivalent amount of water. Although a potential confounding effect of this small amount of water cannot be completely ruled out, its contribution relative to background precipitation is extremely limited, as the total annual N solution input accounts for less than 2% of the annual precipitation at JGS and 1% at SMT, respectively. Moreover, previous studies conducted at these two platforms have shown that this water input did not affect the overall experiment outcomes (Li et al., 2023; Yang et al., 2024; Li et al., 2025b).

### 2.3 Fine root and soil collection

Based on vegetation survey data from both sites and species importance values, six dominant species were identified in JGS, including three trees and three shrubs. In SMT, seventeen dominant species were selected, comprising thirteen trees and four shrubs (**Supplementary Table 2**). The combined importance values of these dominant species exceeded 75%, indicating that they adequately represented the characteristics of the respective communities.

Sampling times were determined based on plant phenological patterns and local climatic conditions at the two study sites. Growing season sampling was conducted in July for the JGS site and in April at SMT, whereas non-growing season sampling was performed in January for JGS and in November at SMT. All field sampling was completed in 2021. Fine roots of dominant species were collected using a combination of root tracing and soil monolith methods, whereby roots were traced from coarse parent roots to fine root segments (Li et al., 2023). To ensure experimental rigor, at least three individual plants were selected for each dominant species, and one soil monolith measuring 30 × 30 × 30 cm (volume = 27,000 cm^3^) was excavated for each individual. All fine roots of the target species with a diameter of ≤ 2 mm were carefully sorted from each soil monolith. Root branching order was determined following the standard morphometric classification system: the most distal, unbranched root tips were defined as first-order roots; roots at the junction of two first-order roots were classified as second-order roots, and so forth, up to fifth-order roots. Broken roots or root segments that could not be fully classified to the fifth order were excluded. This procedure ensured that fine roots from all samples were aligned to the same branching hierarchy and were thus directly comparable (Pregitzer et al., 2002) This sampling protocol typically recovers 60%-80% of total fine root biomass (Grier et al., 1981; Claus and George, 2011).

Community-level fine roots were sampled by randomly selecting three points within each plot and collecting all fine roots within 30 × 30 × 30 cm soil blocks, representing the fine root biomass at the community level. All soil remaining after fine root removal was homogenized, and a 200 g subsample was collected by the quartering method for physicochemical analyses.

### 2.4 Fine root biomass, morphology, stoichiometry, and soil sample measurement

An Epson digital scanner (Epson America, Inc., California, USA) was used to scan fine root images, and root morphological traits were analyzed using Win-RHIZO software (version 2012b, Canada Inc.). After scanning, fine roots were oven dried at 65°C for 48 h to determine biomass, then ground and passed through a 200-mesh sieve. A subsample was analyzed for total C and total N using an elemental analyzer (vario EL cube; Elementar, Langenselbold, Germany). Soil samples were passed through a 200-mesh sieve, homogenized, and divided into two subsamples. One subsample was used to determine soil available N (NH_4_-N and NO_3_-N), and the other was oven dried to a constant weight for measurements of soil pH and soil water content (SWC).

### 2.5 Assessment of FD and CWM of fine root

Fine root functional trait indices (SRL, SRA, TD, root tip density (RTD) root fork density (RFD), specific root tip density (SRTD) and specific root fork density (SRFD)) were calculated according to **Supplementary Table S1**. The species-by-trait (s) matrix was constructed using the morphological and chemical traits of fine roots from dominant species, along with their abundance and distribution within each plot. Functional richness (FRic), functional evenness (FEve), and functional divergence (FDiv) were calculated using Equations 1–8:

(1) $$FRic=\frac{SFic}{Rc}$$

Where *SFic* represents the functional space volume of the observed community (e.g., occupied niche space), and *Rc* denotes the reference volume of functional space defined by the regional species pool.

(2) $$Ew_{I}=\frac{dist\left(i,j\right)}{\left(W_{i}+W_{j}\right)}$$

(3) $$PEw_{I}=\frac{Ew_{I}}{\sum_{i=1}^{S-1}min\left(Ew_{I}\right)}$$

(4) $$FEve=\frac{\sum_{i=1}^{S-1}\min\left(PEw_{I},\frac{1}{S-1}\right)-\frac{1}{S-1}}{1-\frac{1}{S-1}}$$

Where, *dist*(*i*, *j*) represents the Euclidean distance between species *i* and *j*, *W_i_* is the relative biomass of species *i*; *Ew* is the evenness of weights; *I* is the branch length, *Ew_I_* denotes the branch length weighting; and *S* is the number of species.

(5) $$\bar{dG_{I}}=\frac{\sum_{i=1}^{S}dG_{i}}{S}$$

(6) $$\Delta d=\sum_{i=1}^{S}W_{i}\times \left(dG_{i}-\bar{dG_{I}}\right)$$

(7) $$\Delta \left|d\right|=\sum_{i=1}^{S}W_{i}\times \left|dG_{i}-\bar{dG_{I}}\right|$$

(8) $$FDiv=\frac{\Delta d+\bar{dG_{I}}}{\Delta \left|d\right|+\bar{dG_{I}}}$$

Where *S* is the number of species, *W_i_* is the relative biomass of species *i*, $\bar{dG_{I}}$ is the mean Euclidean distance from species *I* to the centroid, *d* denotes the multivariate weighting discretization, Δ*d* represents the abundance weighted deviation of species *I* from the centroid, Δ|d| denotes the absolute abundance weighted deviation.

CWM values were calculated according to Equation 9 below:

(9) $$CWM=\sum_{i=1}^{S}P_{i}\times X_{i}$$

Where *P_i_* denotes the relative abundance of species *i* within a given quadrat, *X_i_* represents the value of a specific functional trait for species *i* in that quadrat.

### 2.6 Statistical analyses and graphic production

ANOVA was used to assess whether N addition way and N addition concentration, and their interaction, significantly influence fine root biomass and soil physicochemical properties across the two locations and the growing season and non-growing season. All statistical significance was set at *p* < 0.05. Data were processed with IBM SPSS Statistics (Version 22.0; SPSS Inc., Chicago, IL, USA), and figures were produced in Origin 2021 (OriginLab, Northampton, MA, USA). Bar plots display means ± standard errors.

FD indices and CWM index were calculated in R (Version 4.5.2) using the vegan package. To explore the influence of variables on community level fine root biomass, Random Forest (RF) model analyses were conducted, with Increase in Node Purity used to rank variable importance and permutation tests used to derive p-values. The top ranked predictors from the RF model results were: Treatment (N addition way and concentration), environmental factors (NH_4_-N, NO_3_-N, pH, and SWC), dominant species (fine root biomass of dominant trees and shrubs), FD indices (FRic, FEve, FDiv), and CWM (In accordance with Li, et al. (2025a), we used PCA to reduce dimensionality and generate composite indices for fine root traits. CWM_1_, representing nutrient absorption, was derived from PCA on CWM values of SRL, SRA, RTD, RFD, SRTD, and SRFD; CWM_2_, representing root lifespan, was similarly derived from an independent PCA on CWM values of TD, Dia, and C/N). To mitigate potential issues from collinearity and nonlinear structure in RF and to compare FD and CWM across growing season and non-growing season, we extracted the top predictor for environment, FD, and CWM and built two hierarchical linear models with environmental controls: FD model= Environment + FD; CWM model = Environment + CWM. Model comparison was based on Adjusted R^2^, calculated as $Adjusted\;R^{2}=1-\frac{\left(1-R^{2}\right)\left(n-1\right)}{n-k-1}$. A bootstrap (n = 1000) was used to obtain the distribution of Adjusted R^2^. Welch’s t test and the Wilcoxon rank sum test compared whether the FD and CWM distributions differed significantly. Significance was based on bootstrap distributions rather than single model results. Before interpretation, standard diagnostic plots (Residuals vs Fitted, Q-Q, Scale Location, and Residuals vs Leverage) were used to assess linearity, residual normality, and homoscedasticity, and VIFs were calculated to ensure no severe multicollinearity (VIF< 5). Analyses were conducted in R using the packages randomForest, rfPermute, performance, boot, and ggplot2 for RF modeling, assessment, and plotting.

## 3 Results

### 3.1 Effects of N addition of fine root biomass

**Figure 2:**
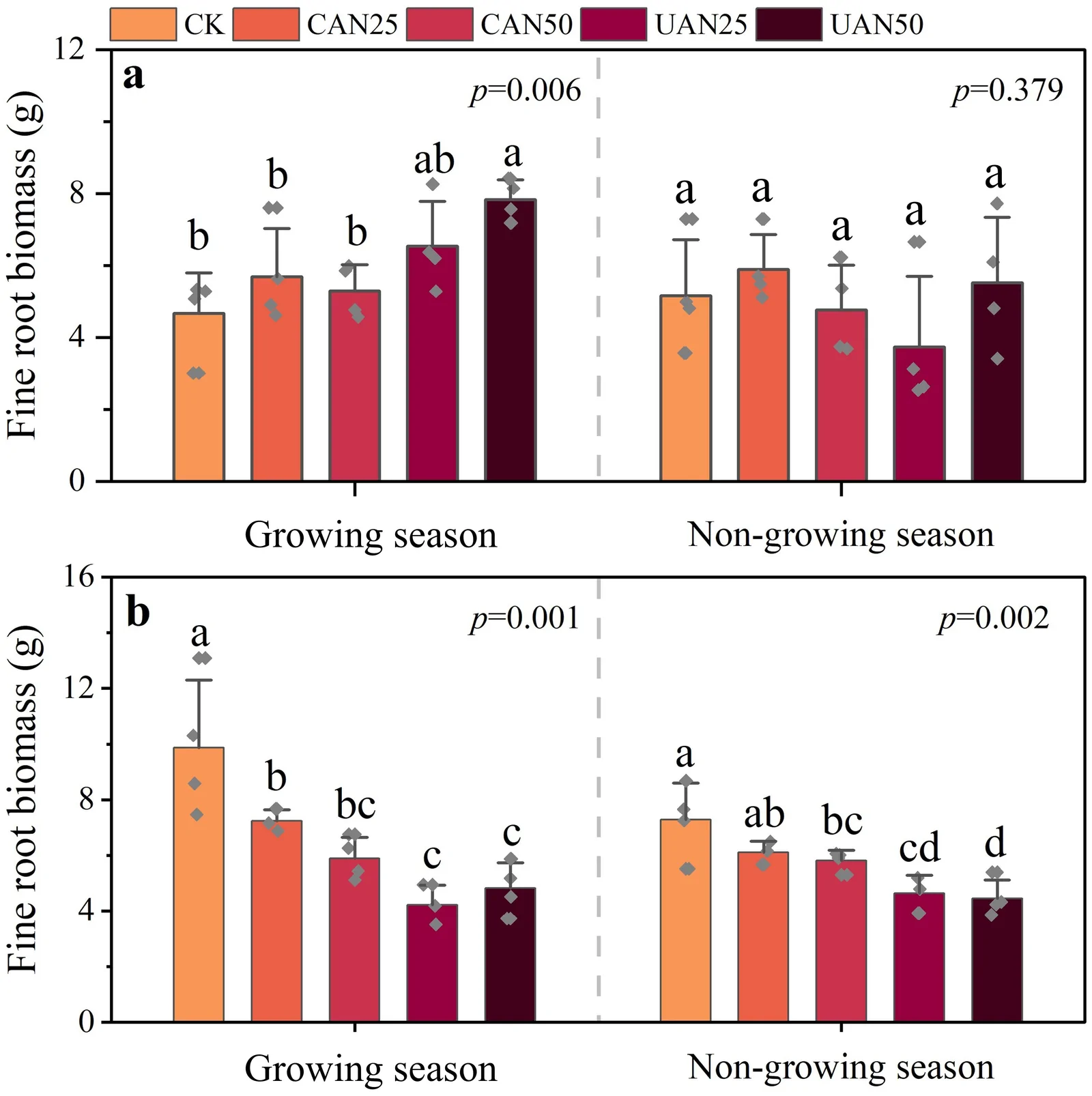
Community-level fine root biomass under different nitrogen (N) addition treatments in the warm-temperate forest **(a)** and subtropical forest **(b)** during the growing and non-growing seasons. Treatments include control (CK), canopy addition N at 25 kg N ha^-1^ yr^-1^ (CAN25), canopy addition N at 50 kg N ha^-1^ yr^-1^ (CAN50), understory addition N at 25 kg N ha^-1^ yr^-1^ (UAN25), and understory addition N at 50 kg N ha^-1^ yr^-1^ (UAN50). Gray diamonds indicate individual data points for each replicate. Different lowercase letters above bars indicate significant differences among treatments within each group at *p* < 0.05. The vertical dashed line separates the two seasons.

In the warm-temperate forest during the growing season, N addition significantly increased community-level fine root biomass (*p* < 0.01, **Figure 2a**), and UAN yielded larger gains than CAN, with growing rates of 39.91% for UAN25 and 67.35% for UAN50, respectively. In the non-growing season, N addition had no significant effect on fine root biomass (**Figure 2a**). NAW, NAC, and the NAW × NAC interaction had no significant effect on fine root biomass (**Table 1**).

**Table 1:** Effects of nitrogen (N) addition way, N addition concentration, and the interaction between N addition way and concentration on community fine root biomass in a warm-temperate (JGS) and subtropical (SMT) forests during growing and non-growing season.

Site	Period	NAW		NAC		NAW × NAC	
		F	*p*	F	*p*	F	*p*
JGS	growing season	0.191	0.675	0.122	0.737	1.386	0.278
	Non-growing season	2.053	0.202	0.035	0.858	1.334	0.292
SMT	growing season	7.73	*	0.69	0.438	1.392	0.283
	Non-growing season	10.478	*	0	0.985	0.055	0.82

This table presents the results of two-way ANOVA. NAW, N addition way, NAC, N addition concentration, NAW × NAC represents the interaction between N addition way and N addition concentration. F denotes the F statistic from the ANOVA, and *p* denotes the significance probability. Asterisks indicate *p* < 0.05.

In the subtropical forest, N addition significantly reduced community-level fine root biomass in both the growing season and the non-growing season (*p* < 0.01, **Figure 2b**). The magnitude of reduction differed significantly by N addition way in both periods, with UAN causing larger declines than CAN. Specifically, the largest decrease occurred with UAN25 during the growing season (−57.17%), and with UAN50 during the non-growing season (−37.56%, **Table 1**). Moreover, in the non-growing season, both CAN and UAN reduced fine root biomass with increasing concentration.

### 3.2 The random forest model shows that the factors affecting the fine root biomass of the community exhibit significant climate zone and seasonal dependence

**Figure 3:**
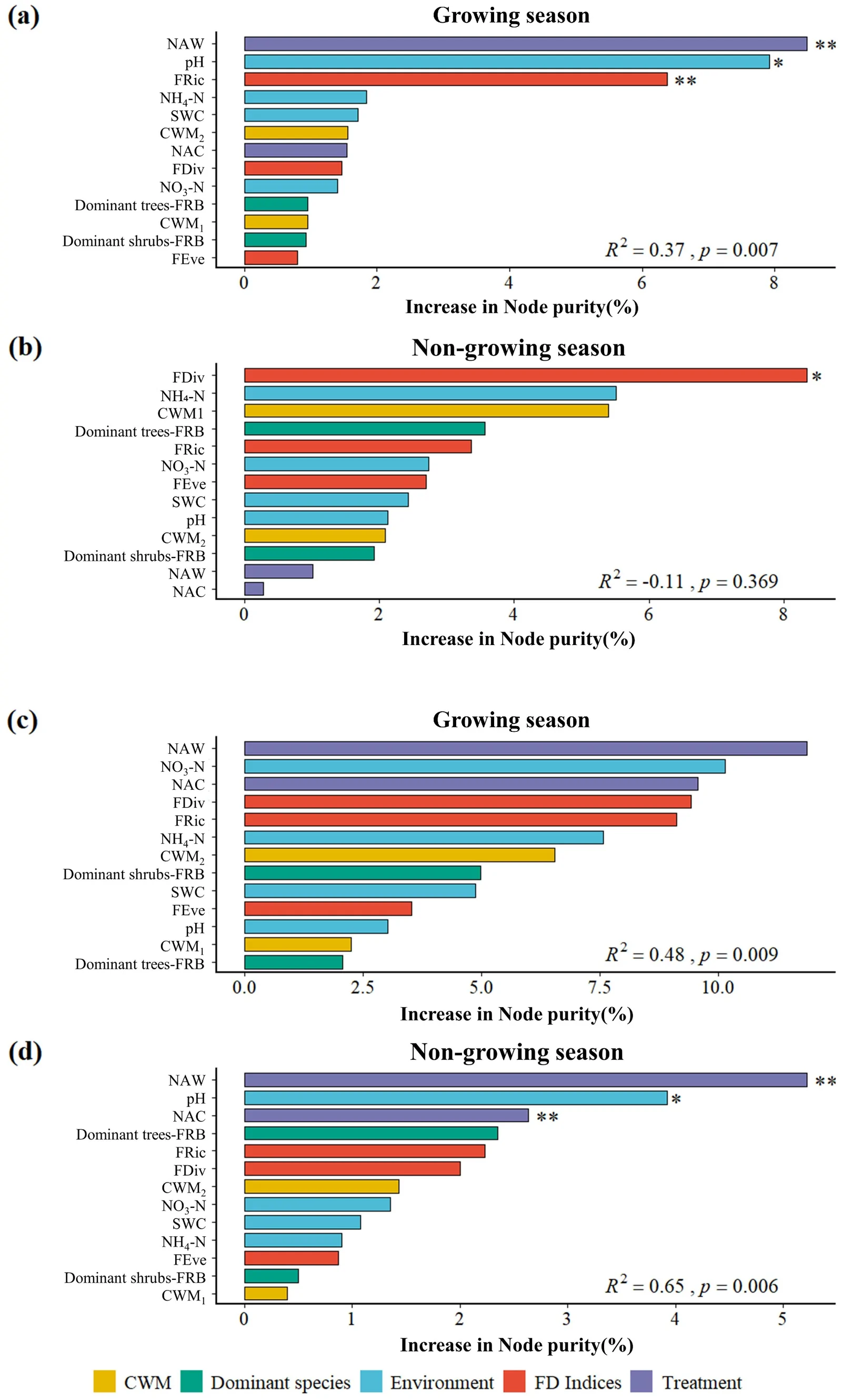
Random forest model analysis of the growing and non-growing seasons in the warm-temperate forest **(a, b)** and subtropical forest **(c, d)**. Figures **(a)** and **(b)** show the importance ranking (increase in node purity) of environmental variables, community weighted mean (CWM), functional diversity (FD), fine root biomass of dominant species, and N addition treatments in the random forest model for the growing season and non-growing Season, respectively. Figures **(c)**, **(d)** show the importance ranking of the random forest model for the subtropical forest for growing season and non-growing season, respectively. NAW, N addition way. NAC, N addition concentration. SWC denotes Soil water content The length of each bar represents the contribution of the corresponding predictor to model performance. Asterisks (*) and (**) denote statistical significance at *p* < 0.05 and *p* < 0.01, respectively. Model explanatory power (*R*^2^) and its *p*-values are shown at the bottom right of each figure.

Random forest results show that drivers of fine root biomass exhibit latitudinal differences and pronounced seasonal variation (**Figure 3**). In the warm-temperate forest, the growing season could be predicted by multiple factors, explaining a significant portion of the variation in community-level fine root biomass (R^2^ = 0.37, *p* = 0.007, **Figure 3a**). In contrast, the non-growing season model was not significant and had a negative explanatory power (R^2^ = −0.11, *p* = 0.369, **Figure 3b**). Among the growing season predictors, NAW ranked first and together with pH and FRic significantly explained the variation in community fine root biomass. Among the FD indices, FRic ranked highest and exceeded the top CWM index (CWM_2_) (*p* < 0.01), though the difference was small. However, in the non-growing season, although variables such as FDiv, NH_4_-N, and CWM_1_ ranked highly in importance, the overall model remained non-significant, indicating limited predictive power for the season and possible influence from unmeasured factors (e.g., cold temperature constraints). In the subtropical forest, the results indicated a driver pattern centered on environmental and experimental treatments, with both growing and non-growing seasons showing higher and significant explanatory power (growing Season: R^2^ = 0.48, *p* = 0.009; non-growing season: R^2^ = 0.65, *p* = 0.006). NAW and NAC ranked among the top three across both seasons (**Figures 3c, d**). NO_3_-N and pH were the primary environmental factors in growing season and non-growing season, respectively (*p* < 0.05). FRic and FDiv within the FD set outperformed the top ranked CWM metric (CWM_2_) in all comparisons.

### 3.3 Hierarchical regression model quantifies the differences in explanatory power of FD and CWM on community-level fine root biomass

**Figure 4:**
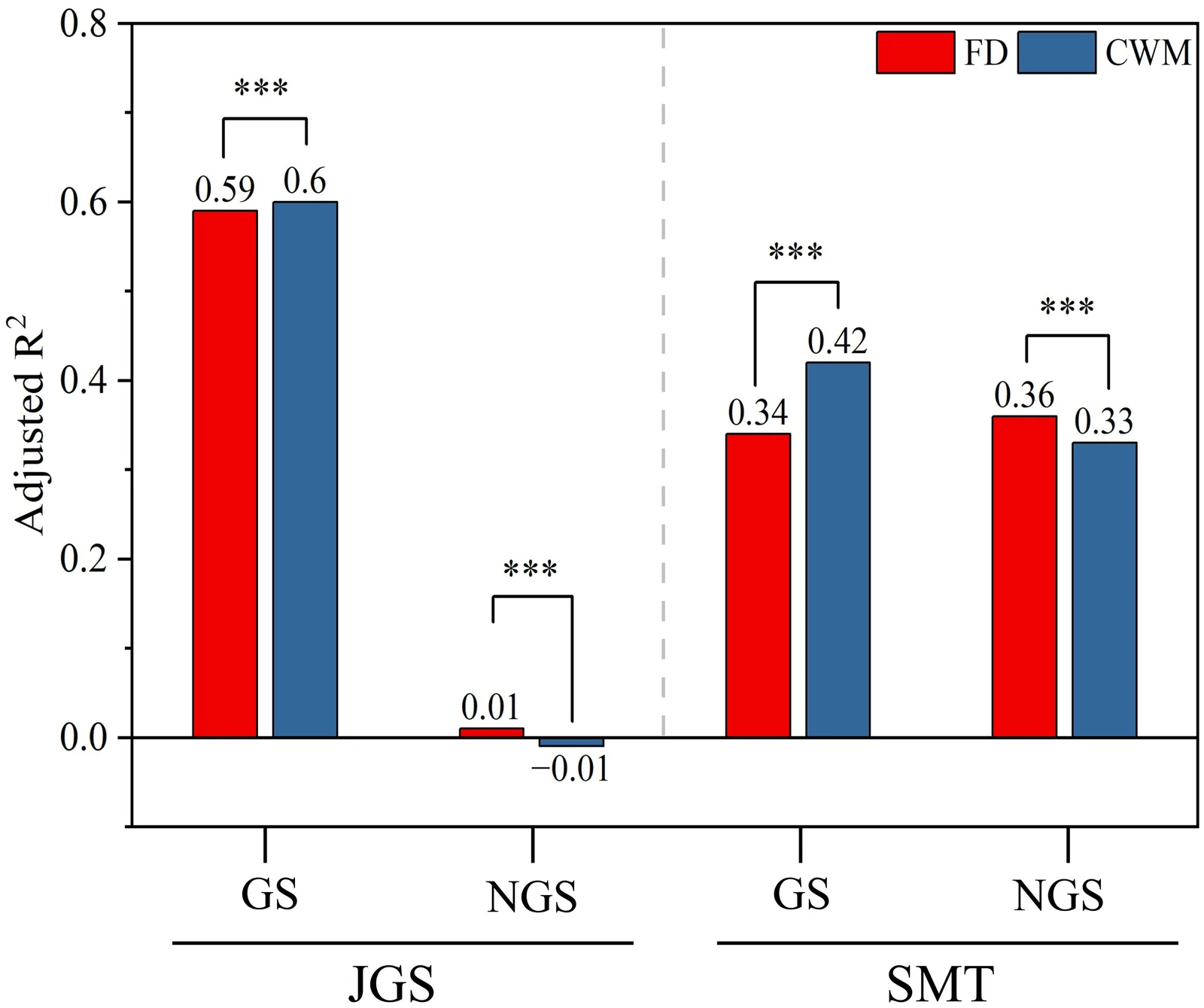
Comparison of explanatory power (Adjusted R^2^) of functional diversity (FD) and community-weighted mean (CWM) derived from hierarchical regression models in warm-temperate (JGS) and subtropical forests (SMT) across the growing season (GS) and non-growing season (NGS). Variables incorporated into the two regression models are described as follows. We extracted the highest-ranked environmental variable, FD metric, and CWM trait for Environment + CWM and Environment + FD hierarchical models at the JGS and SMT sites in both seasons. At the JGS site, the top predictors were pH, FRic and CWM_2_ for the growing season, whereas NH_4_-N, FDiv and CWM_1_ were selected for the non-growing season. Correspondingly, NO_3_-N, FDiv and CWM_2_ dominated the growing season models at the SMT site, while soil pH, FRic and CWM_2_ were prioritized in non-growing season models. Bars denote Adjusted R^2^ values from hierarchical regression models, with CWM and FD indicated in blue and red, respectively. Three asterisks (***) denote significant differences between FD and CWM models at *p* < 0.001. Significance was assessed using bootstrap resampling (1000 iterations) of Adjusted R^2^ to obtain the sampling distribution of model performance, followed by Welch’s t test and the Wilcoxon rank-sum test to evaluate whether FD and CWM differed significantly. Statistical significance reflects differences between the bootstrap distributions rather than single model estimates. JGS and SMT panels are separated by dashed lines. For the specific values of Adjusted R^2^, AIC, BIC, and CV-residuals, please refer to Appendix **Supplementary Table 5**.

Based on hierarchical regression models and bootstrap resampling, results from the warm-temperate forest during the growing season show that CWM (0.60) and FD (0.59) have very similar Adjusted R^2^ values, but the CWM model explains slightly more and more stable variation (*p* < 0.001, **Figure 4**). In the non-growing season, the Adjusted R^2^ for CWM was −0.01, indicating minimal explanatory power, consistent with the RF result (**Figure 3a**), suggesting that CWM did not effectively explain fine root biomass during this period. In contrast, FD (0.10) retained some explanatory power, resulting in a significant difference between the two (*p* < 0.001). The subtropical forest also showed significant differences between FD and CWM in both seasons (*p* < 0.001). In the growing season, CWM (0.42) exceeded FD (0.34), whereas in the non-growing season FD (0.36) exceeded CWM (0.33).

## 4 Discussion

### 4.1 Response of fine root biomass to N addition

The experimental results showed that N addition significantly increased community fine root biomass in the warm-temperate forest during the growing season, but decreased it in the subtropical forest. During the non-growing season, no significant changes were observed in the warm-temperate forest, whereas fine root biomass in the subtropical forest remained significantly reduced. This divergence may reflect differences in background N levels between warm-temperate and subtropical forests (Elrys et al., 2023), which may lead to distinct fine root biomass responses to N addition (**Figure 2**, **S1**). In warm-temperate forests, low ambient N deposition and soil available N levels render plant growth N-limited (Terrer et al., 2019; Li et al., 2021), During the growing season, exogenous N inputs can alleviate this limitation and stimulate belowground resource acquisition; moreover, UAN interacts more directly with the soil surface than CAN. For example, CWM(SRL) and CWM(SRTD) increased markedly (**Supplementary Figure 2**), indicating an increase in fine root surface area and nutrient acquisition efficiency, thereby promoting fine root growth. However, under CAN treatment, part of the added N was intercepted by the canopy and subsequently retained by the canopy itself or taken up by epiphytic organisms and soil microbes (Nair et al., 2016). Therefore, CAN was less effective than UAN in increasing soil available N (Li et al., 2023; Yang et al., 2026) (**Supplementary Table 3**).

During the non-growing season, no significant differences were detected between any N addition treatment and the CK. This may be attributed to the lower winter temperatures in warm-temperate forest, where cold conditions rather than N become the primary limiting factor for plant metabolism and growth, thereby slowing or even halting root turnover, reducing root nutrient and water uptake, and ultimately suppressing fine root growth (Zhang et al., 2025b). In the subtropical forest, N addition significantly suppressed community-level fine root biomass, which may be attributable to further N enrichment reducing the need for C allocation to fine roots; instead, plants may allocate more photosynthates to aboveground tissues (e.g., leaves and stems) to enhance C assimilation and increase leaf biomass (Ghimire et al., 2017; Umaña et al., 2021). Therefore, the decline in fine root biomass is also consistent with the “optimal biomass allocation” theory (Hu et al., 2026). In addition, N addition can lower soil pH and promote acidification (Li et al., 2021), Our results also showed that UAN50 significantly reduced soil pH (**Supplementary Table 4**). In acidic soils, elevated free Al^3+^ can inhibit root tip growth, leading to fine root mortality and reduced biomass (Zhang et al., 2023). Compared with CAN, UAN led to a more pronounced decrease in fine root biomass (**Figure 2b**; **Table 1**). This could be attributed to two factors acting either independently or jointly. First, previous studies have indicated that the forest canopy intercepts 40-80% of the deposited N (Wang et al., 2021), thereby reducing the amount of N reaching the understory soil and mitigating the adverse effects of N saturation on fine roots. Second, leaf stomata may take up a portion of the intercepted N and convert it into organic N, thereby enhancing photosynthesis and reducing reliance on nutrient uptake by fine roots (Nair et al., 2016; Ferraretto et al., 2022). These results indicate that forest canopies may dampen the effects of atmospheric N deposition on belowground processes, and that conventional understory N addition experiments may overestimate the effect of N deposition on forest fine roots.

Although fine root biomass in warm-temperate and subtropical forests responded in opposite directions to N addition, both may be governed by a common regulatory mechanism: the aboveground and belowground C allocation trade-off (Umaña et al., 2021). According to the optimal partitioning theory (Enquist and Niklas, 2002; Hu et al., 2026), N addition increased soil N supply, prompting plants to readjust C allocation to fine roots. This adjustment essentially reflects a balance between resource acquisition and plant demand, and may represent the common driver of fine root biomass changes in the two forest types.

### 4.2 Seasonal differences of FD and CWM between warm-temperate and subtropical forests

Clarifying whether belowground fine root biomass under N deposition is governed primarily by the mass ratio hypothesis or the niche complementarity hypothesis provides a critical basis for understanding the principal drivers of ecosystem functioning under global change. Across both warm-temperate and subtropical forests under long-term N addition, most scenarios showed that N addition way or FD was among the top-ranked variables; in particular, FRic and FDiv frequently rank higher than CWM_1_ and CWM_2_ in importance, although the differences were small (**Figure 3**). By integrating hierarchical regression models with bootstrap-derived adjusted R^2^ values and significance tests, we concluded that the growing season supports the mass-ratio hypothesis, whereas the non-growing season supports the niche complementarity hypothesis (**Figure 4**).

In the warm-temperate forest during the growing season, N addition significantly increased community fine root biomass, with a larger increase under UAN than under CAN (**Figure 2**). The hierarchical regression model showed that both CWM (Adjusted R^2^ = 0.60) and FD (Adjusted R^2^ = 0.59) explain substantial variation, with CWM making a significantly greater contribution (*p* < 0.001, **Figure 4**). This indicates that during the growing season, the mass-ratio effect contributed more strongly to the functioning of warm-temperate forest ecosystems, whereas the niche complementarity effect, although weaker, still provided important functional compensation by enhancing resource use and promoting fine root biomass. In accordance with the mass-ratio hypothesis, dominant species are more effective at occupying space, capturing light, and absorbing nutrients, and their trait values exert a substantial influence on community functioning (Finegan et al., 2015). The expansion of dominant species’ root systems not only increases their own biomass but also enhances resource use efficiency and alters nutrient redistribution, thereby driving an overall increase in community-level fine root biomass. This pattern supports the view that the mass-ratio effect strengthens under resource-rich conditions, provided that the community possesses sufficient functional diversity (Tilman et al., 1997; Grime, 1998; Díaz et al., 2007). The alleviation of N limitation expands the functional space available to the community, and interspecific variation in fine root morphology and functional traits determines the potential and efficiency of resource uptake per unit soil volume; increases in traits such as SRTD and RFD allow deeper and broader exploration and more effective resource capture (**Supplementary Figure 2**), thereby enabling the community to utilize water and nutrients more effectively and promoting fine root production (Freschet et al., 2021; Peng et al., 2026). Therefore, the dynamics of fine root biomass in the warm-temperate forest during the growing season were driven by both the mass-ratio effect and the niche complementarity effect, with the former playing the larger role.

However, in the non-growing season, community-level fine root biomass no longer showed a consistent response to N addition, whereas FD remained significantly more explanatory than CWM (*p* < 0.001). The adjusted R^2^ values were −0.01 for CWM and 0.10 for FD, representing a substantial decline in explanatory power relative to the growing season. This pattern was consistent with the non-growing season results of the random forest analysis (**Figure 3b**). This finding suggests that the non-growing season was governed by niche complementarity, with FD primarily captured by FDiv, indicating that the fine root traits of coexisting species occupied a broader portion of the functional space and represented diverse survival and resource acquisition strategies (Pan et al., 2024). Although root physiology activity is generally reduced in the non-growing season because of low temperatures, species within the community may respond asynchronously to N input across temporal or spatial scales. For example, frost-tolerant species such as *Quercus acutissima* and *Quercus variabilis* may maintain relatively high root activity or flexible resource-use strategies during the non-growing season and may also possess greater nutrient transport capacity or longer root lifespans (Valladares et al., 2014). In contrast, cold-sensitive species may adopt conservative strategies during the non-growing season to reduce root metabolism or enter a state of semi-dormancy, thereby lowering energy expenditure (Zhang et al., 2025b). The diversity of species’ functional strategies and their asynchronous responses to N inputs may enable the community to utilize nutrients more effectively during the non-growing season, thereby driving changes in community fine root biomass (Yachi and Loreau, 1999). In contrast, CWM emphasizes the mean trait values of dominant species; during the non-growing season, these species are often in a maintenance state or semi-dormancy, leading to a reduced direct influence of their traits on fine root biomass. Thus, the non-growing season did not support the mass-ratio hypothesis, but rather highlighted the central role of niche complementarity in sustaining belowground biomass under environmental and seasonal constraints.

In subtropical forest, N addition reduced community-level fine root biomass during both the growing season and the non-growing season, with the way of N addition significantly affecting the biomass (**Figure 2b**; **Table 1**). In the growing season, changes in fine root biomass were mainly driven by the mass ratio effect, whereas those in the non-growing season were governed by niche complementarity. N addition increased soil nutrient levels in the growing season in the subtropical forest (e.g., NH_4_-N and NO_3_-N; **Supplementary Table 3**), driving dominant species to shift along the root economics spectrum and adjust C allocation strategies (Li et al., 2022). When soil available N increases, plants shift from acquisitive to conservative strategies; dominant species allocate more photosynthates to aboveground tissues to compete for light, or to fine roots with faster turnover to increase uptake efficiency rather than maintaining high root biomass (Umaña et al., 2021; Hou et al., 2024). Traits such as CWM(SRL) and CWM(RFD) decreased (**Supplementary Figure 3**). This indicates that the mass-ratio hypothesis helps explain the decline in community-level fine root biomass. In the non-growing season, the niche complementarity effect became the principal driver of changes in fine root biomass. According to biodiversity-ecosystem functioning (BEF) theory (Loreau et al., 2001), more diverse ecosystems exhibit greater stability, because species that are less affected by environmental stress can compensate for those that are reduced, thereby maintaining overall productivity (Luo et al., 2025a). This effect is especially critical during the resource-limited non-growing season. However, in the non-growing season, increased precipitation deficit and soil acidification (**Figure 3d**; **Supplementary Table 4**) shifted community assembly from competition-driven process to environmental filtering. Stress induced by N addition may eliminate species or trait combinations that are poorly adapted to those conditions (Wang et al., 2026a),thereby eroding existing complementary mechanisms and reducing the community’s resource acquisition capacity, ultimately lowering fine root biomass (Loreau and Hector, 2001).

This study demonstrates that across both warm-temperate and subtropical forests, the relative dominance of the mass-ratio hypothesis versus the niche complementarity hypothesis in shaping fine root biomass shows a consistent seasonal reversal: the mass-ratio hypothesis prevails during the growing season, while the niche complementarity hypothesis governs the non-growing season. Previous studies have largely focused on the growing season alone or aboveground processes (Finegan et al., 2015; Chiang et al., 2016; Mason et al., 2020; He et al., 2024), whereas seasonal shifts in belowground processes remain largely underexplored. This finding indicates that the relative contributions of the two hypotheses are not static but vary across seasonal timescales, thus extending the evaluation of these two frameworks from the spatial domain to the temporal dimension.

## 5 Conclusion

Based on a 9-year canopy and understory N addition experiment in warm-temperate and subtropical forests in China, this study combined random forest models and hierarchical regression models to systematically evaluate the effects of N addition on community-level fine root biomass, and extended the mass-ratio hypothesis (driven by CWM) and the niche complementarity hypothesis (driven by FD) to belowground fine root biomass, evaluating their applicability and relative contributions in explaining its variation. N addition significantly increased community-level fine root biomass during the growing season in the warm-temperate forest, whereas no significant response was observed during the non-growing season, likely because of low-temperature limitation and related factors. In the subtropical forest, fine root biomass decreased significantly in both seasons. Across most scenarios, the random forest models identified N addition mode, soil available N, and pH as major drivers, and FD exerted stronger explanatory power than CWM. In both warm-temperate and subtropical forests, seasonal variation in fine root biomass showed a consistent pattern: the growing season was primarily driven by the mass-ratio hypothesis, whereas the non-growing season was governed by the niche complementarity effect. This indicates that the two mechanisms exhibited consistent seasonal shifts across different climate zones and time periods. Future work that comprehensively integrates multidimensional environmental conditions and links aboveground processes with belowground dynamics will help clarify the relative contributions of FD and CWM to the regulation of these ecological processes in forests, thereby refining the theoretical framework underlying the maintenance of forest ecosystem stability.

## Data Availability

The original contributions presented in the study are included in the article/supplementary material. Further inquiries can be directed to the corresponding authors.
