# Supplementary material for: Differential responses of fine root biomass to nitrogen deposition mediated by functional diversity
Source: Front Plant Sci. 2026 Aug 12;17:1884380. doi: 10.3389/fpls.2026.1884380 (PMC13506684; doi:10.3389/fpls.2026.1884380)
Supplement: Supplementary file 1 [file Table1.docx]

**Supplementary Materials**

**Table S1.** Calculation formula for fine root indicators.

| Indicator | Formula | Serial number |
| --- | --- | --- |
| Fine root surface area $(S)$ | $S({cm}^{2})=\pi\sum_{\mathcal{i}=1}^{n} d_{\mathcal{i}}l_{\mathcal{i}}$ | (1) |
| Fine root volume $(V)$ | $V({cm}^{3})=0.25\pi\sum_{\mathcal{i}=1}^{n} d_{\mathcal{i}}^{2}l_{\mathcal{i}}$  In (1) and (2), $l$ is the length of the root, $d$ is the diameter of the root, and $n$ is the number of short root tips. | (2) |
| $SRL(cm/g)$ | $SRL(cm/g)=\frac{l}{M}$ | (3) |
| $SRA({cm}^{2}/g)$ | $SRA({cm}^{2}/g)=\frac{S}{M}$ | (4) |
| $TD(g/{cm}^{3})$ | $TD(g/{cm}^{3})=\frac{M}{V}$ | (5) |
| $RTD(\text{tip/g }root)$ | $RTD(\text{tip/g }root)=\frac{T}{l}$ | (6) |
| $RFD(\text{fork/ groot}\text{)}$ | $RFD(\text{fork/ groot}\text{)}=\frac{F}{l}$ | (7) |
| $SRTD(\text{tip/cm root})$ | $SRTD(\text{tip/cm root})=\frac{T}{M}$ | (8) |
| $SRFD(\text{fork/cm root)}$ | $SRFD(\text{fork/cm root)}=\frac{F}{M}$  In (3-8)，$M$ is fine root biomass，$T$ refers to the number of root tips, $F$ refers to the number of forks. | (9) |

**Table S2.** Dominant species in different forest layers of JGS and SMT forest communities.

| Site | Life form | Species names | Family | Genera |
| --- | --- | --- | --- | --- |
| JGS | Tree | *Quercus acutissima* | Fagaceae | Quercus |
|  |  | *Quercus variabilis* | Fagaceae | Quercus |
|  |  | *Liquidambar formosana* | Hamamelidaceae | Liquidambar |
|  | Shrub | *Lindera glauca* | Lauraceae | Lindera |
|  |  | *Celtis sinensis* | Ulmaceae | Celtis |
|  |  | *Acer buergerianum* | Sapindaceae | Acer |
| SMT | Tree | *Schima superba* | Theaceae | Schima |
|  |  | *Castanea henryi* | Fagaceae | Castanea |
|  |  | *Engelhardia roxburghiana* | Juglandaceae | *Engelhardia* |
|  |  | *Rapanea linearis* | Primulaceae | *Rapanea* |
|  |  | *Machilus chinensis* | Lauraceae | *Machilus* |
|  |  | *Schefflera octophylla* | Araliaceae | *Schefflera* |
|  |  | *Ilex ficoidea* | Aquifoliaceae | *Ilex* |
|  |  | *Cryptocarya chinensis* | Lauraceae | *Cryptocarya* |
|  |  | *Lindera metcalfiana* | Lauraceae | Lindera |
|  |  | *Elaeocarpus dubius* | Elaeocarpaceae | *Elaeocarpus* |
|  |  | *Cryptocarya concinna* | Lauraceae | *Cryptocarya* |
|  |  | *Macaranga sampsonii* | Euphorbiaceae | *Macaranga* |
|  |  | *Elaeocarpus chinensis* | Elaeocarpaceae | *Elaeocarpus* |
|  | Shrub | *Symplocos ramosissima* | Symplocaceae | *Symplocos* |
|  |  | *Blastus cochinchinensis* | Melastomataceae | *Blastus* |
|  |  | *Brassaiopsis glomerulata* | Araliaceae | *Brassaiopsis* |
|  |  | *Aidia canthioides* | Rubiaceae | *Aidia* |

**Table S3.** Effects of nitrogen (N) addition on available nitrogen in warm-temperate and subtropical forest soils during the growing and non-growing seasons.

| Treatment | warm-temperate forest | | subtropical forest | | |
| --- | --- | --- | --- | --- | --- |
|  | NH_4_-N (mg/kg) | NO_3_-N (mg/kg) | NH_4_-N (mg/kg) | | NO_3_-N (mg/kg) |
| growing season | | | | | |
| CK | $\text{7.0±0.61}$ b | $\text{35.35±5.82}$ a | 10.98$\text{±}$0.61 c | 46.48$\text{±}$3.1 c | |
| CAN25 | $\text{7.62±0.97}$ b | $\text{36.75±6.84 a}$ | 12.98$\text{±}$0.62 bc | 52.99$\text{±}$1.16 b | |
| CAN50 | $\text{10.02.±0.79}$ ab | $\text{46.81±6.36}$ a | 13.88$\text{±}$0.63 b | 51.66$\text{±}$1.41 bc | |
| UAN25 | $\text{9.20±2.53}$ ab | $\text{43.38±11.11}$ a | 14.01$\text{±}$0.41 ab | 55.22$\text{±}$1.51 b | |
| UAN50 | $\text{14.36±2.58}$ a | $\text{53.31±10.96}$ a | 16.25$\text{±}$1.36 a | 64.92$\text{±}$1.06 a | |
| non-growing season | | | | | |
| CK | 4.48$\text{±}$1.13 a | $\text{13.33±1.15}$ b | 6.38$\text{±}$0.72 c | 20.22$\text{±}$1.13c | |
| CAN25 | 3.82$\text{±}$0.92 a | $\text{14.34±0.98}$ b | $\text{6.41±0.17}$ c | 23.36$\text{±}$1.09 bc | |
| CAN50 | 4.30$\text{±}$0.79 a | $\text{25.23±2.32}$ a | $\text{8.86±1.15}$ ab | 25.42$\text{±}$1.15 b | |
| UAN25 | 3.04$\text{±}$0.38 a | $\text{21.33±2.81}$ ab | $\text{7.02±0.91}$ b | 22.75$\text{±}$1.87 bc | |
| UAN50 | 3.65$\text{±}$0.71 a | $\text{25.89±4.41 }$a | $\text{10.61±0.95}$ a | 29.75$\text{±}$1.03 a | |

CK denotes the control; CAN25 denotes canopy N addition at 25 kg N ha⁻^1^ year⁻^1^; CAN50 denotes canopy N addition at 50 kg N ha⁻^1^ year⁻^1^; UAN25 denotes understory N addition at 25 kg N ha⁻^1^ year⁻^1^; UAN50 denotes understory N addition at 50 kg N ha⁻^1^ year⁻^1^. Data are presented as means $\pm$ standard error. Lowercase letters (a, b, c) indicate statistically significant differences among treatments for available nitrogen. Comparisons across different study sites, seasons, and indicators were not performed.

**Table S4.** Effects of nitrogen addition on available nitrogen in warm-temperate and subtropical forest soils during the growing and non-growing seasons.

| Treatment | warm-temperate forest | | subtropical forest | |
| --- | --- | --- | --- | --- |
|  | SWC (%) | pH | SWC (%) | pH |
| growing season | | | | |
| CK | 0.235$\text{±}$0.01 bc | 4.23$\text{±}$0.06 a | 0.342$\text{±}$0.01 ab | 4.48$\text{±}$0.1 a |
| CAN25 | 0.259$\text{±}$0.01 a | 4.17$\text{±}$0.02 ab | 0.364$\text{±}$0.02 ab | 4.99$\text{±0}$.16 a |
| CAN50 | 0.258$\text{±}$0.02 ab | 4.16$\text{±}$0.03 ab | 0.401$\text{±}$0.03 a | 4.66$\text{±0.}$41 ab |
| UAN25 | 0.257$\text{±}$0.01 ab | 4.06$\text{±}$0.03 b | 0.311$\text{±}$0.01 b | 4.22$\text{±0}$.51 ab |
| UAN50 | 0.232$\text{±}$0.01 c | 4.07$\text{±}$0.02 b | 0.301$\text{±}$0.04 b | 3.92$\text{±}$0.06 b |
| non-growing season | | | | |
| CK | $0.273\text{±}$0.01 a | 4.11$\text{±}$0.02 a | 0.321$\text{±}$0.05 a | 4.22$\text{±0}$.13a |
| CAN25 | 0.282$\text{±}$0.01 a | 4.05$\text{±}$0.04 a | 0.303$\text{±}$0.03 a | 4.36$\text{±0}$.09 a |
| CAN50 | 0.294$\text{±}$0.01 a | 3.95$\text{±}$0.01 a | 0.331$\text{±}$0.02 a | 4.02$\text{±0}$.15 ab |
| UAN25 | 0.281$\text{±}$0.01 a | $4.13\text{±}$0.03 a | 0.291$\text{±}$0.01 a | 3.95$\text{±0}$.07 ab |
| UAN50 | 0.259$\text{±}$0.01 a | $4.18\text{±}$0.05 a | 0.321$\text{±}$0.06 a | 3.75$\text{±0}$.13 b |

CK denotes the control; CAN25 denotes canopy N addition at 25 kg N ha⁻^1^ year⁻^1^; CAN50 denotes canopy N addition at 50 kg N ha⁻^1^ year⁻^1^; UAN25 denotes understory N addition at 25 kg N ha⁻^1^ year⁻^1^; UAN50 denotes understory N addition at 50 kg N ha⁻^1^ year⁻^1^. SWC denotes Soil water content. Data are presented as means ± standard error. Lowercase letters (a, b, c) indicate statistically significant differences among treatments for SWC and pH. Comparisons across different study sites, seasons, and indicators were not performed.

**Table S5.** Adjusted R², AIC, BIC, and cross-validated residuals (CV-residuals) from hierarchical regression models using functional diversity (FD) and community-weighted mean traits (CWM) to explain community-level fine-root biomass across warm-temperate (JGS) and subtropical (SMT) forests during the growing season (GS) and non-growing season (NGS).

| Site | Season | Mechanism | Adjust R^2^ | AIC | BIC | CV-Residuals |
| --- | --- | --- | --- | --- | --- | --- |
| JGS | GS | FD | 0.5889 | 43.6823 | 47.6653 | 0.9506 |
|  |  | CWM | 0.5991 | 43.2278 | 47.2107 | 0.9103 |
|  | NGS | FD | 0.0960 | 59.4885 | 63.4714 | 2.5625 |
|  |  | CWM | ﹣0.0124 | 61.7532 | 65.7361 | 3.8396 |
| SMT | GS | FD | 0.3400 | 48.3220 | 51.8835 | 1.5871 |
|  |  | CWM | 0.4200 | 45.9948 | 49.5563 | 1.2948 |
|  | NGS | FD | 0.3616 | 47.7216 | 51.2831 | 1.3958 |
|  |  | CWM | 0.3322 | 48.5325 | 52.0940 | 1.4365 |

**Figure S1.** Dominant trees and shrubs fine root biomass under different nitrogen (N) addition treatments in the warm-temperate (JGS) and subtropical (SMT) forests during the growing and non-growing seasons. Figures (a, b) and (c, d) show fine root biomass for the JGS forest in the growing season and non-growing season, respectively, while figures (e, f) and (g, h) show corresponding results for the SMT forest. Treatments include control (CK), canopy N addition at 25 kg N ha⁻^1^ yr⁻^1^ (CAN25), canopy N addition at 50 kg N ha⁻^1^ yr⁻^1^ (CAN50), understory N addition at 25 kg N ha⁻^1^ yr⁻^1^ (UAN25), and understory N addition at 50 kg N ha⁻^1^ yr⁻^1^ (UAN50). Different lowercase letters above bars indicate significant differences among treatments within each group at *p* $<$ 0.05 based on one-way ANOVA test. Error bars represent standard error.

**Figure S2.** Effects of canopy and understory nitrogen (N) addition on community weighted mean (CWM) fine root traits in warm-temperate forest (JGS). Treatments include control (CK), canopy N addition at 25 kg N ha⁻^1^ yr⁻^1^ (CAN25), canopy N addition at 50 kg N ha⁻^1^ yr⁻^1^ (CAN50), understory N addition at 25 kg N ha⁻^1^ yr⁻^1^ (UAN25), and understory N addition at 50 kg N ha⁻^1^ yr⁻^1^ (UAN50). Figures (a) and (b) show the changes in CWM (nutrient absorption) and CWM (lifespan) in the growing season. Figures (c) and (d) show the changes in CWM (nutrient absorption) and CWM (lifespan) in the non-growing season. CWM (nutrient absorption) includes the fine root functional traits of specific root length (SRL), specific root area (SRA), root tip density (RTD), root fork density (RFD), specific root tip density (SRTD) and specific root fork density (SRFD). CWM (lifespan) includes the fine root functional traits of total C/ total N (C/N), root tissue density (TD) and diameter (Dia). Solid lines indicate mean values and error bars represent standard errors. Asterisks denote significant differences at *p* $<$ 0.05.

**Figure S3.** Effects of canopy and understory nitrogen (N) addition on community weighted mean (CWM) fine root traits in subtropical forest (SMT). Treatments include control (CK), canopy N addition at 25 kg N ha⁻^1^ yr⁻^1^ (CAN25), canopy N addition at 50 kg N ha⁻^1^ yr⁻^1^ (CAN50), understory N addition at 25 kg N ha⁻^1^ yr⁻^1^ (UAN25), and understory N addition at 50 kg N ha⁻^1^ yr⁻^1^ (UAN50). Figures (a) and (b) show the changes in CWM (nutrient absorption) and CWM (lifespan) in the growing season. Figures (c) and (d) show the changes in CWM (nutrient absorption) and CWM (lifespan) in the non-growing season. CWM (nutrient absorption) includes the fine root functional traits of specific root length (SRL), specific root area (SRA), root tip density (RTD), root fork density (RFD), specific root tip density (SRTD) and specific root fork density (SRFD). CWM (lifespan) includes the fine root functional traits of total C/ total N (C/N), root tissue density (TD) and diameter (Dia). Solid lines indicate mean values and error bars represent standard errors. Asterisks denote significant differences at *p* $<$ 0.05.

**Figure S4.** Comparison of functional diversity metrics across warm-temperate and subtropical forests in growing season (GS) and non-growing season (NGS). FEve donates functional evenness. FDiv donates functional divergence. FRic donates functional richness. CK (control), CAN25 (canopy N addition 25 kg N ha⁻^1^ year⁻^1^), CAN50 (canopy N addition 50 kg N ha⁻^1^ year⁻^1^), UAN25 (understory N addition 25 kg N ha⁻^1^ year⁻^1^), UAN50 (understory N addition 50 kg N ha⁻^1^ year⁻^1^). Colors distinguish forest type and season. Letters a, b, c above bars indicates significant differences among treatments at *p* $<$ 0.05. Solid lines indicate mean values and error bars represent standard errors.
